# Supplementary material for: Partograph utilization and associated factors among obstetric care givers in governmental health institutions of Jigjiga and Degehabur towns, Somali region, Ethiopia: A cross-sectional study
Source: PLoS One. 2022 Mar 9;17(3):e0264373. doi: 10.1371/journal.pone.0264373 (PMC8906604; doi:10.1371/journal.pone.0264373)
Supplement: Attachment — Submitted filename: Second review.pdf [file pone.0264373.s004.pdf]

PLOS ONE

Partograph utilization and associated factors among obstetric care givers in governmental health institutions of Jigjiga and Degehabur towns, Somali region, Ethiopia: a cross-sectional study

--Manuscript Draft--

**Manuscript Number:**	PONE-D-21-20057R1
**Article Type:**	Research Article
**Full Title:**	Partograph utilization and associated factors among obstetric care givers in governmental health institutions of Jigjiga and Degehabur towns, Somali region, Ethiopia: a cross-sectional study
**Short Title:**	Photograph utilization of obstetric care givers in health facilities of Jigjiga and Degehabur towns, Ethiopia
**Corresponding Author:**	Liyew Mekonen Ayehubizu, MPH<br>Jigjiga University<br>Jigjiga, ETHIOPIA
**Keywords:**	Partograph; Utilization; Obstetric care givers; Governmental health institutions; Somali region; Ethiopia
**Abstract:**	Background : Partograph is a simple, inexpensive & economical tool that provides a continuous graphical overview of labour and prevents prolonged and obstructed labor. We aimed to assess partograph utilization and associated factors among obstetric care givers in governmental health institutions of Jigjiga and Degehabur Towns, Somali Region, Ethiopia.<br>Methods: An institution based cross-sectional quantitative study was carried out among obstetric care givers who were working in governmental health institutions. Systematic random sampling with proportional to size allocation was used to recruit a total of 235 study participants. Self-administered questionnaire was used to collect data in this study. Three data collectors and one supervisor were recruited and trained to facilitate the data collection activities. Data were entered into Epi data software and exported into SPSS (23.0) for analysis. Descriptive statistics, bivariate and multivariate logistic regression were computed to determine proportions and significant association with partograph utilization among obstetric care givers.<br>Result: Less than half of obstetric care givers, 41 %(95%CI: 34.5- 46.9) had good partograph utilization to monitor progress of labor. Being female[AOR =2.364, 95%CI:(1.027-5.441)], availability of partograph[AOR=4.633, 95%CI: (1.698-12.640)], having good knowledge [AOR=6.902, 95%CI:(2.620-18.183)], receiving on job training [AOR= 15.463, 95%CI:(6.948-34.416)] and positive attitude towards partograph [AOR=2.986, 95%CI:(1.250-7.136)] were significantly associated with partograph utilization.<br>Conclusion: Partograph utilization in this study was low. Especial emphasizes and interventions should be given to periodic on job training that improve knowledge and attitude of obstetric care givers to increase partograph utilization.
**Order of Authors:**	Liyew Mekonen Ayehubizu, MPH
	Abebe Tadesse Tibebeu
	Metsihet Tariku Fetene
	Semehal Haile Yohannes
**Response to Reviewers:**	Comments raised by both reviewers and editor are important to enrich this research article. Authors have also looked each comments and modified the document accordingly.
**Additional Information:**	
**Question**	**Response**
**Financial Disclosure**	The author(s) received no specific funding for this work.

Powered by Editorial Manager® and ProduXion Manager® from Aries Systems Corporation

Enter a financial disclosure statement that describes the sources of funding for the work included in this submission. Review the submission guidelines for detailed requirements. View published research articles from *PLOS ONE* for specific examples.<br><br>This statement is required for submission and **will appear in the published article** if the submission is accepted. Please make sure it is accurate.<br><br>**Unfunded studies**<br>Enter: *The author(s) received no specific funding for this work.*<br><br>**Funded studies**<br>Enter a statement with the following details:<br>• Initials of the authors who received each award<br>• Grant numbers awarded to each author<br>• The full name of each funder<br>• URL of each funder website<br>• Did the sponsors or funders play any role in the study design, data collection and analysis, decision to publish, or preparation of the manuscript?<br>• **NO** - Include this sentence at the end of your statement: *The funders had no role in study design, data collection and analysis, decision to publish, or preparation of the manuscript.*<br>• **YES** - Specify the role(s) played.<br><br>* typeset	
**Competing Interests**<br><br>Use the instructions below to enter a competing interest statement for this submission. On behalf of all authors, disclose any competing interests that could be perceived to bias this work—acknowledging all financial support and any other relevant financial or non-financial competing interests.<br><br>This statement is required for submission and **will appear in the published article** if the submission is accepted. Please make	The authors have declared that no competing interests exist.

sure it is accurate and that any funding sources listed in your Funding Information later in the submission form are also declared in your Financial Disclosure statement.<br><br>View published research articles from *PLOS ONE* for specific examples.<br><br>**NO authors have competing interests**<br><br>Enter: *The authors have declared that no competing interests exist.*<br><br>**Authors with competing interests**<br><br>Enter competing interest details beginning with this statement:<br><br>*I have read the journal's policy and the authors of this manuscript have the following competing interests: [insert competing interests here]*<br><br>* typeset	
**Ethics Statement**<br><br>Enter an ethics statement for this submission. This statement is required if the study involved:<br><br>• Human participants<br>• Human specimens or tissue<br>• Vertebrate animals or cephalopods<br>• Vertebrate embryos or tissues<br>• Field research<br><br>Write "N/A" if the submission does not require an ethics statement.<br><br>General guidance is provided below. Consult the submission guidelines for detailed instructions. **Make sure that all information entered here is included in the Methods section of the manuscript.**	Before conducting this study, the ethical clearance letter was sought from the Jigjiga University college of Medicine and health sciences school of graduate studies research and ethics Committee. Then an official letter was obtained from the Jigjiga University College of Medicine and health sciences, school of graduate studies and submitted to Jigjiga and Degehabur Town health offices. The purpose of the study was explained and informed consent was obtained from each respondent before collection of data.

Powered by Editorial Manager® and ProduXion Manager® from Aries Systems Corporation

**Format for specific study types**<br><br>**Human Subject Research (involving human participants and/or tissue)**<br>• Give the name of the institutional review board or ethics committee that approved the study<br>• Include the approval number and/or a statement indicating approval of this research<br>• Indicate the form of consent obtained (written/oral) or the reason that consent was not obtained (e.g. the data were analyzed anonymously)<br><br>**Animal Research (involving vertebrate animals, embryos or tissues)**<br>• Provide the name of the Institutional Animal Care and Use Committee (IACUC) or other relevant ethics board that reviewed the study protocol, and indicate whether they approved this research or granted a formal waiver of ethical approval<br>• Include an approval number if one was obtained<br>• If the study involved *non-human primates*, add *additional details* about animal welfare and steps taken to ameliorate suffering<br>• If anesthesia, euthanasia, or any kind of animal sacrifice is part of the study, include briefly which substances and/or methods were applied<br><br>**Field Research**<br><br>Include the following details if this study involves the collection of plant, animal, or other materials from a natural setting:<br>• Field permit number<br>• Name of the institution or relevant body that granted permission	
**Data Availability**<br><br>Authors are required to make all data underlying the findings described fully available, without restriction, and from the time of publication. PLOS allows rare exceptions to address legal and ethical concerns. See the PLOS Data Policy and FAQ for detailed information.	Yes - all data are fully available without restriction

Powered by Editorial Manager® and ProduXion Manager® from Aries Systems Corporation

A Data Availability Statement describing where the data can be found is required at submission. Your answers to this question constitute the Data Availability Statement and **will be published in the article**, if accepted.<br><br>**Important:** Stating 'data available on request from the author' is not sufficient. If your data are only available upon request, select 'No' for the first question and explain your exceptional situation in the text box.<br><br>Do the authors confirm that all data underlying the findings described in their manuscript are fully available without restriction?	
**Describe where the data may be found in full sentences. If you are copying our sample text, replace any instances of XXX with the appropriate details.**<br><br>• If the data are **held or will be held in a public repository**, include URLs, accession numbers or DOIs. If this information will only be available after acceptance, indicate this by ticking the box below. For example: *All XXX files are available from the XXX database (accession number(s) XXX, XXX.*).<br>• If the data are all contained **within the manuscript and/or Supporting Information files**, enter the following: *All relevant data are within the manuscript and its Supporting Information files.*<br>• If neither of these applies but you are able to provide **details of access elsewhere**, with or without limitations, please do so. For example:<br><br>*Data cannot be shared publicly because of [XXX]. Data are available from the XXX Institutional Data Access / Ethics Committee (contact via XXX) for researchers who meet the criteria for access to confidential data.*<br><br>*The data underlying the results presented in the study are available from (include the name of the third party*	All relevant data are within the manuscript and its Supporting Information files.

Powered by Editorial Manager® and ProduXion Manager® from Aries Systems Corporation

and contact information or URL).<br>• This text is appropriate if the data are owned by a third party and authors do not have permission to share the data.<br><br>* typeset	
Additional data availability information:	

Manuscript

Click here to access/download;Manuscript;Manuscript.docx

Partograph utilization and associated factors among obstetric care givers in governmental health institutions of Jigjiga and Degehabur towns, Somali region, Ethiopia: a cross-sectional study

Abebe Tadesse[1], Liyew Mekonen[2], Metsihet Tariku[3] and Semehal Haile[4]

Abstract

Background: Partograph is a simple, inexpensive & economical tool that provides a continuous graphical overview of labour and prevents prolonged and obstructed labor. The purpose of the study is to assess partograph utilization and associated factors among obstetric care givers in governmental health institutions of Jigjiga and Degehabur Towns, Somali Region, Ethiopia.

Methods: An institution based cross-sectional quantitative study was carried out among obstetric care givers who were working in governmental health institutions. Systematic random sampling with proportional to size allocation was used to recruit a total of 235 study participants. Self-administered questionnaire was used to collect data in this study. Three data collectors and one supervisor were recruited and trained to facilitate the data collection activities. Data were entered into Epi data software and exported into SPSS (23.0) for analysis. Descriptive statistics, bivariate and multivariate logistic regression were computed to determine proportions and significant association with partograph utilization among obstetric care givers.

Result: Less than half of obstetric care givers, 41[illegible]%(95%CI: 34.5- 46.9) had good partograph utilization to monitor progress of labor. Being female[AOR =2.364, 95%CI:(1.027-5.441)], availability of partograph[AOR=4.633, 95%CI: (1.698-12.640)], having good knowledge [AOR=6.902, 95%CI:(2.620-18.183)], receiving on job training [AOR= 15.463, 95%CI:(6.948-34.416)] and positive attitude towards partograph [AOR=2.986, 95%CI:(1.250-7.136)] were significantly associated with partograph utilization.

Conclusion: Partograph utilization in this study was low. Especial emphasizes and interventions should be given to periodic on job training that improve knowledge and attitude of obstetric care givers to increase partograph utilization.

Key words: Partograph, Utilization, Obstetric care givers, Governmental health institutions, Somali Region, Ethiopia

[1]Department of Midwifery, College of Medicine and Health Sciences, Jigjiga University

[2]Department of Public Health, College of Medicine and Health Sciences, Jigjiga University

[3]Department of Nursing, College of Medicine and Health Sciences, Jigjiga University

[4]Department of Neonatal Nursing, College of Medicine and Health Sciences, Jigjiga University

Background

Women are suffering many health problems during their pregnancy as well as at the time of child birth/delivery; globally, there were an estimated number of 15% expected births that developed life-threatening complications during pregnancy, delivery or the postpartum period **(1-4)**.

In 2017, about 295 000 women died during and following pregnancy and childbirth. Sub-Saharan Africa alone accounted for roughly two-thirds (196 000) of maternal deaths, while Southern Asia accounted for nearly one-fifth (58 000); and both accounted for approximately 86% (254 000) of the estimated global maternal deaths(5).According to the 2015 Ethiopia Demographic and Health Survey, maternal mortality ratio was 353 per 100,000 live births in Ethiopia **(6-9)**. Though it was slightly increased to 412 in 2016 **(10)**. Maternal mortality was still high in 2019 accounted to 412 per 100,000 live births **(11)**.

The major causes of maternal and neonatal death in Ethiopia are obstructed labour and prolonged labour which was account for (13%) of maternal deaths (9, 12). Obstructed and prolonged labour contribute to series of complication in mothers and newborn infants if it is not managed early(13). These complications can be prevented by proper utilization of partograph during the progress of labour and providing skill delivery services(14).

The partograph is a graphical presentation of the progress of labour, and of fetal and maternal condition during labour (2, 12, 15-17). It consists of four sections: the maternal information, the fetal conditions record, the labor progress record, and the maternal conditions record (17-19). The fetal condition record may track fetal heart rate, amniotic liquor, and molding of the fetal skull. The labor progress record tracks cervical dilatation and descent of the fetus' head over time, comparing it to a pre-printed "alert" and "action" lines (2, 9, 15, 18). The maternal conditions record often captures contractions, blood pressure, pulse, urine output, temperature, and drugs administered including drugs to help the uterus contract (12, 17-20).

Universal utilization of the partograph during labour is recommended by world health organization since routine use of partograph is helpful to make better decisions for the diagnosis and management of prolonged and obstructed labour (17) . It is still not broadly

used in the developing countries especially in Africa include Ethiopia. Studies done in Baghdad, a metropolitan area in Ghana and Lusaka: in Zambia, showed that58%, 54% and 87.5% of the participants used partograph to monitor progress of labour respectively (21-23). In Ethiopia, there is no consistent use of the partograph during labour; Studies done in Asella referral and teaching hospital, Sidama zone, Bale zone, East Gojjam zone, Addis Ababa city administration; showed that 26%, 50.7%, 70.2%, 53.85% and 69% of the participants used partograph to monitor progress of labour respectively(1, 9, 14-16). This inconsistent use of partograph due to different factors such as participant's sex, age, year of clinical service, health profession, knowledge about partograph, attitude towards partograph, absence of training on partograph, work place and work over load(1, 2, 12, 14, 15, 17, 21, 24).

Assessing obstetric care giver's practical utilization of partograph and its determinants has great value to design appropriate intervention strategies to provide quality maternity care. Therefore, the purpose of this study was to assess utilization of partograph and associated factors among obstetric care givers working in governmental health institutions of Jigjiga and Degehabur towns, Somali region, Ethiopia.

Methods

Study setting and period

The study was conducted in public health institutions Jigjiga and Degehabur towns from May18/2020 – July 8/2020. Jigjiga town, a capital city of Somali Region, is located 635kms east of Addis Ababa. It has 20 kebeles. The town administration has two hospitals, two health centres and fourteen health posts. In the town there are 1,208 clinical health professional workers (212 midwives, 11 HO, 137 medical doctors and 6 Gynecologist and obstetrician) that work in obstetric care at governmental health institutions. It has 366 total numbers of obstetric care givers.

Degehabur is a town in the Somali Region of Ethiopia, is located 800 &165 kms north of Addis Ababa and Jigjiga town respectively. It is the administrative centre of Degehabur woreda. The town administration has 1 hospital and 1 health centre of public health institutions. It has also 258 clinical health professional workers. Among those 51 midwives, 9 Health officer , 10 medical doctors, 1 Emergency Surgery and 1 obstetrician that work in obstetric care in the town of public health institutions. It has 72 total numbers of obstetric

care givers. Total number of obstetric care givers in two towns is 438 that work public health institutions.

Study design

An institution based cross-sectional study design was conducted to assess utilization of partograph and associated factors.

Study population

All obstetric care providers (midwives, nurses, general practitioners, and health offers working in governmental health institutions) in Jigjiga and Degehabur Towns, Somali Region, Ethiopia included in the study.

Sample size determination

The sample size was estimated using single proportion formula by assuming 5% marginal error and 95% confidence interval (σ= 0.05) and study conducted in central zone, Tigray in which the proportion of utilization of partograph among obstetric care givers were 73.3% (2). By adding 10% for non-respondents the final sample size was taken as 196. Moreover, the double population proportion formula was used to determine the sample size for factors associated with partograph utilization to get maximum possible sample size. It was calculated for some of the associated factors obtained from different literatures using Epi info statistical software version 7 with the following assumptions: confidence level = 95%, power = 80%, the ratio of unexposed to exposed almost equivalent to 2 and partograph utilization among those do not get additional training is 56.5% with AOR 2.4(14).Therefore, the final estimated sample size is 235 after adding 10% non response rate which was higher than the sample size calculated using single population proportion formula.

Sampling procedure

From all health institutions found in Jigjiga and Degehabur towns, six health institutions (Jigjiga university Sheik Hassen Yabare Referal Hospital, Kharamara Hospital, Ablele health center, Ayerdega health center, Degehabour General Hospital, and Degehabour health center) were selected using a simple random sampling technique.

The total number of health professionals found in selected hospitals and health centers in Jigjiga town were 234 and 50 respectively. Additionally, 51 health professionals from the Degehabour General hospital and 21 from Degehabour health center were included. The required number of study subjects was calculated via proportional allocation, with 187 from Jigjiga and 48 from Degehabour. Study subjects were chosen using a simple random sampling procedure.

Data collection instrument and techniques

Semi - structured questionnaire which was developed from different literatures was used (2, 16, 17, 21, 22, 24). It was prepared in English version. Three data collectors and one supervisor were hired during the data collection period.

Data processing and analysis

Data was entered, checked, and analyzed using SPSS version 23.0. Descriptive statistics was employed to calculate frequencies, median and percentage. Attitude was measured as follows: there were six (6) attitude determining Likert scale type questions prepared to assess respondents' attitude towards partograph. The responses of "strongly agree was scored 5, agree was scored 4, uncertain was scored 3, disagree was scored 2 & strongly disagree was scored 1. The scoring was reversed for negative statements. The scores of the items were summed-up and the total was divided by the number of the items, giving a mean score. These score was converted into a percent score and mean were computed. The attitude was considered "positive" if the scored 60% or more and "negative" if less than 60%.

Bivariable logistic regression analysis was made using OR and 95% CI to assess the association of independent variable with the outcome separately. Based on Bivariate analysis variables that showed significant association at ($p < 0.2$) were entered to multivariable analysis to select Predictor variables of factors affecting partograph utilization. Finally, variables that showed significant association at ($p < 0.05$) were identified as independent predictors of partograph utilization.

Ethical considerations

The Ethical approval was obtained from Institutional Review Board of College of Health Sciences, Jigjiga University on May 14/2020 with reference number RERC/016/2020.

Communications with relevant bodies was made through a formal letter obtained from regional health. The objective and importance of the study was explained to the study participants. Data was collected after full informed written consent was obtained from participants. Confidentiality of the information and privacy is maintained.

Results

Socio-demographic characteristics of study participants

Two hundred twenty eight respondents completed all the questionnaires correctly making a response rate of 97.02%. About 70.6% of obstetric care givers were females. The median ages of the respondents were 26.00 (±3 IQR) years and more than half 56.1% of them were within the age group 25-29 years. Regarding their profession, 66.7 % were midwives followed by medical practitioner (MD) 13.6 %. Regarding to participant's working place, 71.1% of them were working at hospitals and the rest 25.0% were working in health centers. More than two third of the respondents 68.4% had ≤4 clinical service years and median year of clinical service of obstetric care givers were 3.00 (±3 IQR) years (Table 1).

Table 1: Socio-demographic characteristics of obstetric care givers in governmental health institutions of Jigjiga and Degehabur Towns, Somali region, Ethiopia, 2020 (n=228)

Variables	Frequency	Percent
**Sex**		
Male	67	29.4
Female	161	70.6
**Age group**		
20-24	54	23.7
25-29	128	56.1
30 and older	46	20.2
**Professional qualification**		
Nurse	28	12.2
Midwife	152	66.7
HO (health officer)	17	7.5
General practitioner (MD)	31	13.6
**Year of service**		
≤4	156	68.4
5-10	55	24.1
≥11	17	7.5

Place of work		
Health center	66	28.9
Hospital	162	71.1

Knowledge of obstetric care givers about partograph

All participants had heard about partograph while 52.6% of them know the exact definition of partograph. Regarding to starting time of plot on partograph, 83.3% of the participants correctly identified the exact time when to start plotting partograph. However, 61.4% of the respondents did not know the component of partograph and also 71.5%, 68%, 76.8% of the respondents did not know the definition of alert line, action line and satisfactory of labour progress respectively. Pertaining to use of partograph, most of the participant 78.9% mentioned that partograph should be used for all women in active first stage of labor. Majority (63.6%) of respondents said they had not received any photographs on the job training (Table 2).

Table 2: Knowledge of obstetric care givers about partograph in governmental health institutions of Jigjiga and Degehabur Towns, Somali region, Ethiopia, 2020 (n=228)

Variables	Frequency	Percent
**Heard about partograph**		
Yes	228	100.0
**Definition of partograph**		
A tool to be used only in active phase of labor	120	52.6
A graphic method of recording first stage of labor	71	31.2
A salient feature of recording the whole process of labor	37	6.2
**Component of the partograph**		
List all	88	38.6
Not list all	140	61.4
**Knowledge about the start time of plotting partograph**		
At 4cm cervical dilatation	190	83.3
At 3cm cervical dilatation	18	7.9
When labor is diagnosed	20	8.8
**Definition of alert line**		
Define correctly	65	28.5
Not define correctly	163	71.5
**Definition of action line**		
Define correctly	73	32

Not define correctly	155	68
**Definition of satisfactory of labor progress**		
Define correctly	53	23.2
Not define correctly	175	76.8
**Detected any complication using a partograph**		
Yes	196	86
No	32	14
**List one complication if you detect using partograph**		
List	180	96.8
Not list	16	8.2
**Type of client that needs partograph use**		
Primgravid	17	7.5
Multiparious	16	7
Eclamptic patients	15	6.6
All women in active labour	180	78.9
**On job training**		
Yes	83	36.4
No	145	63.6

Regarding respondents' knowledge about partograph, only less than one third of them 28.5% had good knowledge (Figure 1).

Attitude of obstetric care givers towards partograph utilization

Less than half of them (46%) strongly agreed that the photograph is beneficial. More than one third of participants, 45.2% of them were agreed that partograph is favorable as it alerts obstetric care givers of any deviation from normal and 43.9% of the respondents agreed that health care givers are able to identify problems and recognize complications early. Less than one third (26.3%) of them agreed that skilled birth attendant must use a partograph for every labouring mother. Near to thirty one percent (30.7%) of them were uncertain and 28.5% were strongly disagreed that using partograph enables health care givers perform essential basic interventions. And also 35.1% of them were strongly disagreed that using partograph misleads management as the progress of labour and the partograph alert line are not aligned in most pregnant women (Table 3).

Table 3: Attitude of obstetric care givers towards partograph utilization governmental health institutions of Jigjiga and Degehabur Towns, Somali region, Ethiopia, 2020 (n=228)

Variables	Frequency	Percent
**Partograph is beneficial**		
Strongly agree	105	46
Agree	95	41.7
Uncertain	13	5.7
Strongly disagree	15	6.6
**Partograph is very favorable as it alerts health workers any deviation from normal**		
Strongly agree	50	22
Agree	113	49.6
Uncertain	45	19.7
Disagree	14	6.1
Strongly disagree	6	2.6
**By using partograph, health care providers are able to identify problems and recognize complications early**		
Strongly agree	31	13.6
Agree	100	43.9
Uncertain	70	30.7
Disagree	14	6.1
Strongly disagree	13	5.7
**Skilled birth attendant must use partograph on every laboring mother**		
Strongly agree	26	11.4
Agree	60	26.3
Uncertain	90	39.5
Disagree	39	17.1
Strongly disagree	13	5.7
**Using partograph enables health care givers perform essential basic interventions and make referrals to appropriate levels of care when necessary**		
Strongly agree	10	4.4
Agree	23	10.1
Uncertain	70	30.7
Disagree	60	26.3
Strongly disagree	65	28.5
**Using partograph misleads management as the progress of labour and the partograph alert line are not aligned in most pregnant woman**		
Strongly agree	16	7
Agree	22	9.7
Uncertain	50	21.9
Disagree	60	26.3
Strongly disagree	80	35.1

Regarding respondents' attitude towards partograph utilization, only less than one third of them (18%) had positive attitude (Figure 2).

Reasons for not using the partograph

As indicated in figure 3, less than half (42.5%) of respondents said that lack of orientation was the reason for not using the partograph when monitoring women in labor.

Partograph utilization

In this study, majority (74.1%) of the respondents had reported the availability of partograph in the facility. More than half (64%) of the participants were partially recorded parameters of partograph on the tool. Similarly, (26.8%) of the participants were conducted deliveries from 7 to 9 in the institution per shift day. Most (92.1%) of the participants reported that they hadn't faced any problem to utilize partograph. Majority (68.4%) of participants had work load in their facility. More than three-fourths (76.3%) gave advice for the obstetric care givers to encourage use partograph during labor (Table 4).

Table 4: Utilization of partograph by obstetric care givers in governmental health institutions of Jigjiga and Degehabur Towns, Somali region, Ethiopia, 2020 (n=228)

Variables	Frequency	Percent
**Availability of partograph**		
Yes	169	74.1
No	59	25.9
**Partograph use currently**		
Yes	199	87.3
No	29	12.7
**Frequency of partograph utilization**		
Routinely	71	31.1
Sometimes	128	56.1
Occasionally	29	12.7
**Components of partograph plotted**		
Complete	80	35.1
Incomplete	148	64.9
**Number of deliveries conducted per shift day**		
≤ 3	60	26.3
4 to 6	55	24.1

7 to 9	61	26.8
10 and above	52	22.8
**Reasons for not utilizing partograph during labor**		
Lack of orientation	97	42.5
Availability of other methods	24	10.5
Lack of commitment	61	28.8
Lack of supervisions	46	20.2
**Facing any problem to utilize the partograph**		
Yes	18	7.9
No	210	92.1
**Work load**		
Yes	185	81.1
No	43	18.9
**Suggestion in order to encourage partograph utilization**		
Give suggestion	174	76.3
Not give suggestion	54	23.7

Overall from 228 participants, 93(41%) of the participants were had good partograph utilization (Figure 4).

Factors associated with partograph utilization

Bivariate analysis was performed to select candidate variables for multivariable analysis. Accordingly, sex of participant (being female)[COR=2.149, 95%CI: (1.162-3.975)], working in Hospital[COR=1.714, 95%CI: (0.937-3.135)], having good knowledge [COR=2.417, 95%CI: (1.244-4.696)], availability of partograph [COR= 6.451, 95%CI: (2.887-14.414)], taking on job training[COR=12.075, 95%CI: (6.343-22.988)] and positive attitude towards partograph [COR=2.734, 95%CI: (1.365-5.477)] were among factors showed that candidate variables for multivariate analysis at p value</= 0.2.

Multivariate analysis was performed for the purpose of controlling the multiple confounding effects of variables. Accordingly, sex of participant (being female) [AOR =2.364, 95%CI:(1.027-5.441)], availability of partograph[AOR=4.633, 95%CI: (1.698-12.640)], having good knowledge [AOR=6.902, 95%CI:(2.620-18.183)], receiving on job training [AOR=15.463, 95%CI:(6.948-34.416)] and positive attitude towards partograph [AOR=2.986, 95%CI:(1.250-7.136)] were factors showing significant association with partograph utilization at p-value</= 0.05 (Table 5).

Table 5: Bivariate and multivariable analysis of factors associated with partograph utilization of obstetric care givers in governmental health institutions of Jigjiga and Degehabur Towns, Somali region, Ethiopia, 2020 (n=228).

**Variables**	**Partograph utilization**		**COR**	**AOR (95%CI)**	**p-value**
	**Yes No (%)**	**No No (%)**			
**Sex**					
Female	70()	76()	2.363(1.322-4.224)	2.364(1.027-5.411)*	0.043
Male	23()	59()	1	1	
**Work place**					
Hospital	74(45.1)	90(54.9)	1.714(0.937-3.135)	1.347(0.575-3.154)	0.492
Health center	21(31.8)	45(68.2)	1	1	
**Availability of partograph**					
Yes	85(50.3)	84(49.7)	6.451(2.887-14.414)	4.633(1.698-12.640)**	0.003
No	8(13.6)	51(86.4)	1	1	
**Knowledge about partograph**					
Good	35(53.8)	30(46.2)	2.417(1.244-4.696)	6.902(2.620-18.183)***	0.000
Satisfactory	30(39.0)	47(61.0)	1.322(0.695-2.515)	2.674(1.066-6.712)*	0.036
Poor	28(32.6)	58(67.4)	1	1	
**On job training**					
Yes	63(75.9)	20(24.1)	12.075(6.343-22.988)	15.463(6.948-34.416)***	0.000
No	30(20.7)	115(79.3)	1	1	
**Attitude towards partograph utilization**					
Positive	25(61.0)	16(39.0)	2.734(1.365-5.477)	2.986(1.250-7.136)*	0.014
Negative	68(36.4)	119(63.6)	1	1	

Significant at *p-value<0.05, **p-value <0.01 and ***p-value <0.001

DISCUSSION

The proportion of partograph utilization was 41% (95%CI: 34.6-46.9). This finding is inconsistent with study conducted in Baghdad (58%), Metropolitan area in Ghana (54%), Bale zone (70.2%), North Showa Zone (81.1%) , West Showa Zone (84.6%) and Central Tigray Zone (73.3%) (2, 12, 16, 17, 21, 22). The lack of well-designed and integrated programs, such as mentorship and supportive supervision, could be the cause of this difference. Other factors that may contribute to lower partograph utilization include a lack of expertise, a lack of understanding, insufficient partograph training, and a negative attitude among study participants.

This finding is higher than the study conducted in Asella, Teaching and Referral Hospital, Ethiopia (1). These discrepancies may be due to the sample size, different study area and time of study.

Findings of this study also indicated that positively significant association between sex and partograph utilization. Female obstetric care givers were more than two times more likely to good partograph utilization than males. This finding is consistent with a previous study done in Bale zone, Ethiopia (16). It is understandable that the majority of birth attendants assigned to the labor ward on the study area are female midwives who have had the opportunity to participate in partograph usage trainings, which have increased their knowledge and abilities.

Availability of the partograph in health facility is also associated with its utilization. Those health care givers who had partograph tools in their institution were more than four times more likely to use partograph than had not partograph in their facility. This finding is consistent with a previous study done in Asella, Ethiopia and West Showa Oromia, Ethiopia (1, 12). This implies that the availability of partograph by itself is essential to motivate obstetrics giver for use of this tools starting from active first stage of labour.

Findings of this study also indicated that significant association between knowledge and partograph utilization. Those obstetric care givers who had good knowledge about partograph were more than six times more likely to good partograph utilization than their counterparts. This finding is consistent with a previous study conducted in Enugu Metropolis, North Showa Zone, Ethiopia, West Showa Zone, Oromia, Ethiopia and Bale zone (12, 16, 17, 24). The possible reason might be Obstetrics care givers who have knowledge on purpose of partograph may use it more frequently to prevent prolonged labour and related complications.

This research finding showed that on-job training on partograph had a significant association with partograph utilization. Those obstetric care givers who received on job training on partograph were more than fifteen times more likely to utilize partograph than who had not received on- job training. This finding is supported by the study conducted in West Showa zone Oromia Ethiopia, Addis Ababa city administration, Bale zone, East Gojjam zone, central Tigray zone, Easter Ethiopia, North Showa zone and Sidama zone (2, 9, 12, 14-17, 20).On job trainings enhance knowledge ; and improve attitude and skills which in turn increase partograph utilization.

Those obstetric care givers who had positive attitude towards partograph were three times more likely to utilize partograph than to their counterparts. This finding is consistent with a previous study done in North Showa Zone, Ethiopia (17). The possible reason might be those who had good attitude toward the use of partograph might be committed to improve their skill in use.

CONCLUSION AND RECOMMENDATIONS

This study revealed that the proportion of partograph utilization by the study participants was low according to Modified WHO partograph and previous studies. Being female obstetrics care giver, availability of partograph, having good knowledge about partograph, having on job training and having positive attitude of participants towards partograph were significantly associated with partograph utilization.

To Jigjiga and Degehabur towns health offices

Sustainable and accessible supply of partograph sheets should be available to all institutions to maintain consistency of its utilization.

To stake holders

Provision of periodic on-job training on partograph is mandatory to improve knowledge and attitude of obstetric care givers towards partograph utilization.

To obstetric care givers

Partograph should be used for all laboring women and taken seriously by the care givers and it should be considered as a tool for diagnosing problems, perform essential basic interventions and make referral to appropriate levels of care when necessary during the progress of labour.

To researchers

Further researchers have to be done using qualitative research methodology and on private health institutions to get a comprehensive picture.

Abbreviations

ANC: Ante Natal Care; AOR: Adjusted Odds Ratio; APGAR: Activity, Pulse, Grimace, Appearance &Respiration; CI: Confidence Interval; COR: Crude Odds Ratio; CSA:
Central Statistical Agency; EDHS: Ethiopian Demographic and Health Survey; FHR: Fetal Heart Rate; ICM: International Council of Midwives; IMB: Integrated Model of Behavior; MCH: Maternal and Child Health; MM: Maternal Mortality; MMR Maternal Mortality Rate; SD: Standard Deviation; SMI: Safe Motherhood Initiatives; SPSS: Statistical Package for Social Sciences; UN: United Nation; UNFPA: United Nations Population Fund Affairs; UNICEF: United Nations Children Fund; USA: United State of America; WHO: World Health Organization

Acknowledgment

We appreciate Jigjiga University, College of medicine and Health Sciences, School of Graduate Studies, department of Maternity Nursing for giving us this opportunity. We would like to thank Karamara hospital administration office, Jigjiga University Shuik Hassan Yabare Referral Hospital, Jigjiga City health office and Degehabur town health office staffs; for giving base line information for this study. Finally, we would like to thank data collectors for the contribution of data collection and we would like to thank participates that voluntary involve in this study. We would also like to extend our appreciation to those who has helped me a lot in giving additional advice.

Authors' contributions

AT designed the research, analyzed the data, interpreted the findings and drafted the manuscript. LM and MT involved in all process of this research.SH reviewed a manuscript. All authors read and approved the final manuscript for publication.

Funding

Not applicable.

Availability of data and materials

The datasets generated and/or analyzed during the current study are fully available.

Consent for publication

Not applicable.

Competing interests

There is no competing interest among the authors.

Author details

[1]College of Medicine and Health sciences, department of Midwifery, Jigjiga University, Jigjiga, Ethiopia. [2] College of Medicine and Health sciences, department of Midwifery, Jigjiga University, Jigjiga, Ethiopia. [3] College of Medicine and Health sciences, department of Midwifery, Jigjiga University, Jigjiga, Ethiopia

References

1. Bekele D, Beyene K, Hinkosa L, Shemsu MN. Partograph Utilization and Associated Factors among Graduating Health Professional Students in Asella Referal and Teaching Hospital, Ethiopia, 2016. STM Journal. 2016;6(2).
2. Hailu T, Nigus K, Gidey G, Hailu B, Moges Y. Assessment of partograph utilization and associated factors among obstetric care givers at public health institutions in central zone, Tigray, Ethiopia. BMC research notes. 2018;11(1):710.
3. Melese KG, Bekiru ET. Knowledge of partograph utilization and its associated factors: A cross-sectional survey in Wolaita Zone Southern Ethiopia. International Journal of Nursing and Midwifery. 2019;11(1):1-6.
4. Muthusi UM, Nyamoita MG, Stephen M. Contribution of Completed Modified World Health Organization Partograph on Maternal and Foetal Mortality Reduction in Health Facilities in Makueni County, Kenya. Health. 2019;5(6):227-36.
5. World Health O. Trends in maternal mortality 2000 to 2017: estimates by WHO, UNICEF, UNFPA, World Bank Group and the United Nations Population Division. Geneva: World Health Organization; 2019 2019.
6. Alkema L, Chou D, Hogan D, Zhang S, Moller A-B, Gemmill A, et al. Global, regional, and national levels and trends in maternal mortality between 1990 and 2015, with scenario-based projections to 2030: a systematic analysis by the UN Maternal Mortality Estimation Inter-Agency Group. The Lancet. 2016;387(10017):462-74.
7. Federal Democratic Republic of Ethiopia Ministry of Health (FMOH) EPHI. National maternal death surveillance and response annual report 2008 EFY. FMOH, EPHI. 2017.
8. WHO U, UNFPA, and The World Bank and the United Nations Population Division. Trends in Maternal Mortality. World Health Organization. 2015.
9. Zelellw DA, Tegegne TK. Level of partograph utilization and its associated factors among obstetric caregivers at public health facilities in East Gojam Zone, Northwest Ethiopia. PLoS One. 2018;13(7):e0200479.
10. Agency CS. Census, in 2015. Central Statistical Agency and ICF International. 2015.
11. Indicators K. Mini Demographic and Health Survey. EPHI and ICF. 2019.
12. Willi W. Magnitude of Partograph Use and Factors that Affecting Its Utilization Among Obstetric Caregivers in Public Health Institutions of West Showa Zone, Oromia Regional State, Ethiopia 2015: Addis Ababa University; 2015.

13. Bosomprah S, Tatem AJ, Dotse- Gborgbortsi W, Aboagye P, Matthews Z. Spatial distribution of emergency obstetric and newborn care services in Ghana: Using the evidence to plan interventions. International Journal of Gynecology & Obstetrics. 2016;132(1):130-4.
14. Hagos AA, Teka EC, Degu G. Utilization of Partograph and its associated factors among midwives working in public health institutions, Addis Ababa City Administration, Ethiopia, 2017. BMC Pregnancy and Childbirth. 2020;20(1):49.
15. Kidist Eshetu EH, Dubale Dulla. Magnitude of Partograph Use and Associated Factors among Obstetric Care Givers in Public Health Institution in Sidama Zone, Southern Ethiopia. Diversity and Equality in Health and Care (2017) 14(6): 316-323 2017;14(6):316-23.

16. Markos D, Bogale D. Knowledge and utilization of partograph among health care professionals in public health institutions of Bale zone, Southeast Ethiopia. Public health. 2016;137:162-8.
17. Wakgari N, Amano A, Berta M, Tessema GA. Partograph utilization and associated factors among obstetric care providers in North Shoa Zone, Central Ethiopia: a cross sectional study. African health sciences. 2015;15(2):552-9.
18. Angasu K. Assessment of partograph utilization and associated factors among obstetric care providers in public health facilities in Jimma town, Jimma zone, Oromia region , South West Ethiopia, 2016. BMC Pregnancy Childbirth 2016;60(2).

19. Chemeda G, Teklewold B, Daka K. Partograph Utilization and Factors Associated with Poor Perinatal Outcomes in Wolaita Sodo University Referral Hospital, Southern Ethiopia. Journal of Health, Medicine and Nursing. 2019;63.
20. Mezmur H, Semahegn A, Tegegne BS. Health professional's knowledge and use of the partograph in public health institutions in eastern Ethiopia: a cross-sectional study. BMC Pregnancy Childbirth. 2017;17(1):291.
21. Abdul-Saheb TM, Al-Deen LD. Knowledge and Use of Partograph among Obstetric Care Givers in a Sample of Hospitals in Baghdad-2017. Iraqi Journal Of Community Medicine 2017;30(3):115-22.

22. Opoku BK, Nguah SB. Utilization of the modified WHO partograph in assessing the progress of labour in a metropolitan area in Ghana. Research Journal of Women’s Health. 2015;2(2):1-7.
23. Victoria Mwiinga Kalusopa SM, Mutinta Muleya, Kutemba Lyangenda, Maureen Sianga, Francesca Msiska, Carol Porteous, Concepta Kwaleyela, Alice Ngoma Hazemba and Margaret Maimbolwa. Use of the partograph among midwives at University Teaching Hospital, Lusaka: a LAMRN project in Zambia. AFRICAN JOURNAL OF MIDWIFERY AND WOMEN’S HEALTH. 2017;11(2).
24. Nwaneri A, Ndie E, Ehiemere I, Okafor E, Ezenduka P. Evaluation of Factors Affecting the Utilization of Partograph by Nurses/Midwives in Primary/Secondary Health Facilities in Enugu Metropolis. Gynecol Obstet (Sunnyvale). 2017;7(446):2161-0932.1000446.
25. Bekiru KGMaET. Knowledge of partograph utilization and its associated factors: A cross-sectional survey in Wolaita Zone Southern Ethiopia. International Journal of Nursing and Midwifery. January 2019;Vol. 11(1):pp. 1-6.

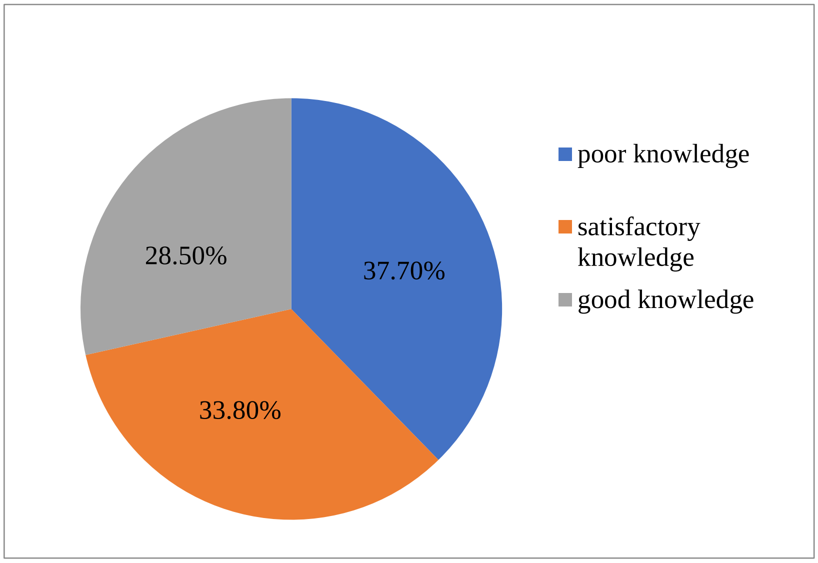

Figure 1: Knowledge of obstetric care givers about pantograph in governmental health institutions of Jigjiga and Degehabur Towns, Somali region, Ethiopia, 2020 (n=228)

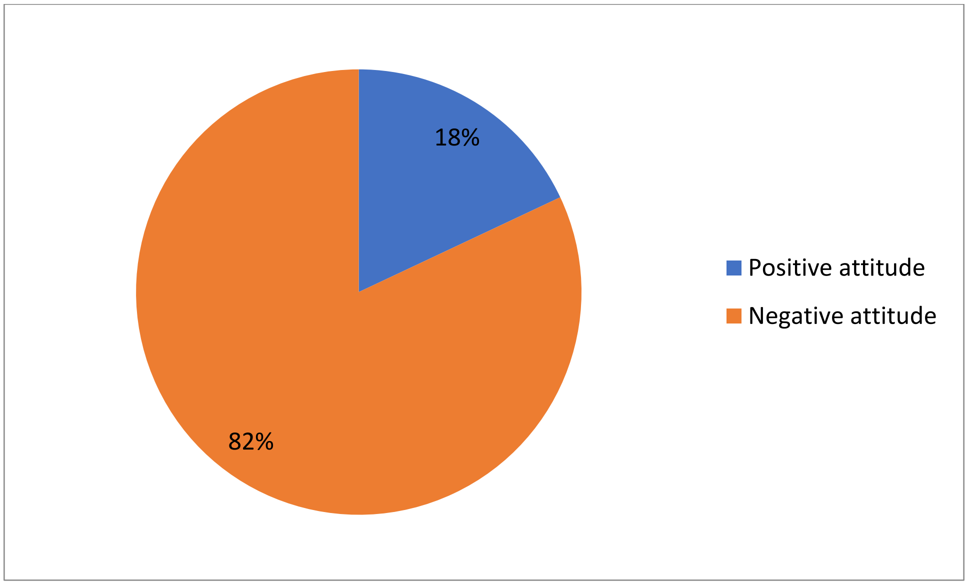

Figure 2: Attitude of obstetric care givers towards partograph utilization governmental health institutions of Jigjiga and Degehabur Towns, Somali region, Ethiopia, 2020 (n=228).

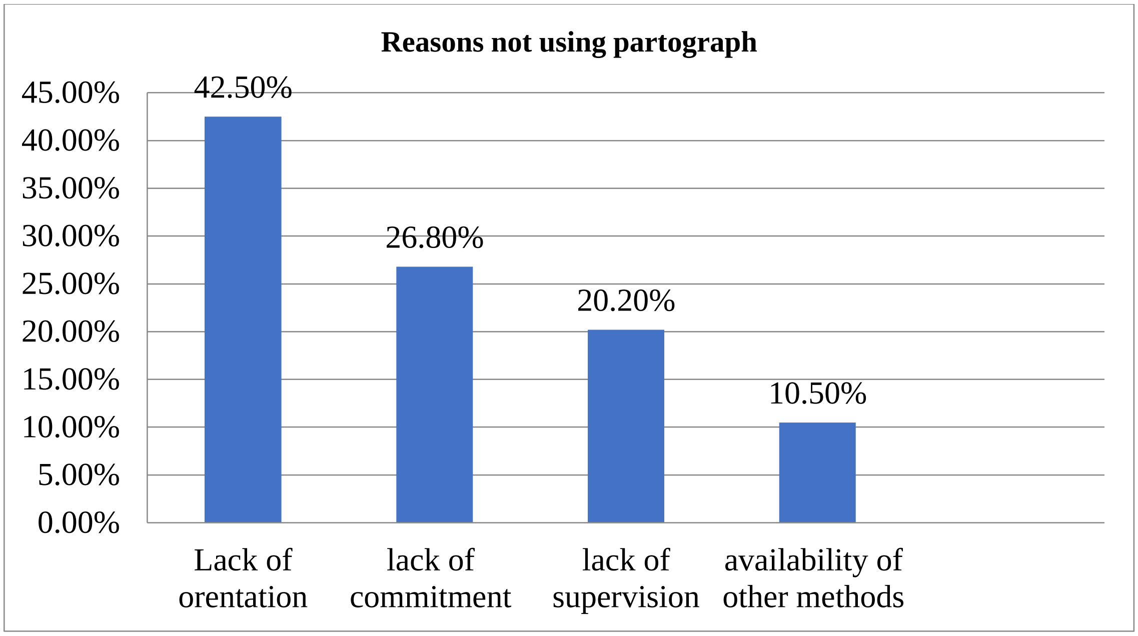

Figure 3: Reasons for not using the partograph by obstetric care givers in governmental health institutions of Jigjiga and Degehabur Towns, Somali region, Ethiopia, 2020 (n=228)

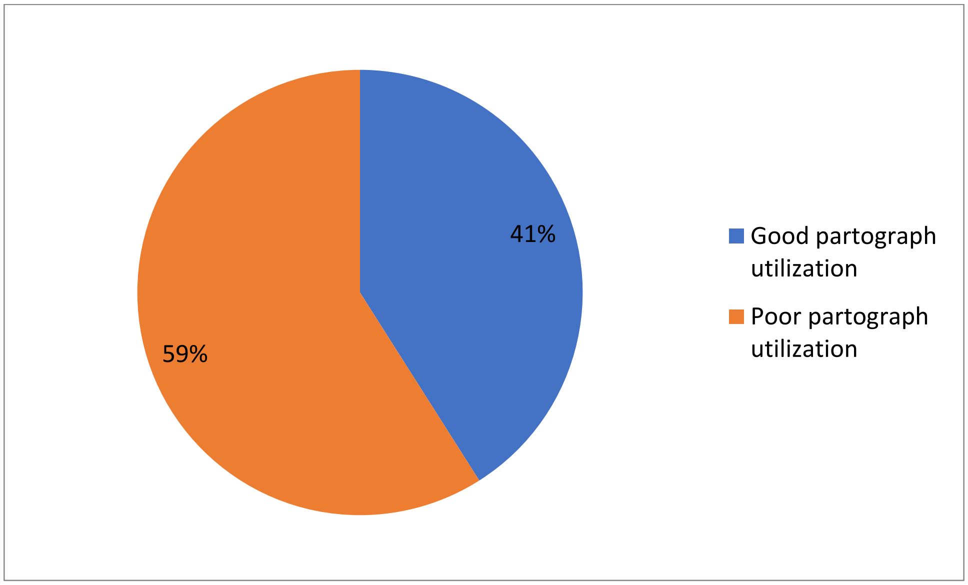

Figure 4: Utilization of partograph by obstetric care givers in governmental health institutions of Jigjiga and Degehabur Towns, Somali region, Ethiopia, 2020 (n=228).

Ethical clearance letter

Click here to access/download
Supporting Information
Ethical Clerance letter.pdf

Partograph SPSS file

Click here to access/download
Supporting Information - Compressed/ZIP File Archive
Partograph SPSS file.rar

Partograph utilization and associated factors among obstetric care givers in governmental health institutions of Jigjiga and Degehabur towns, Somali region, Ethiopia: a cross-sectional study

Abebe Tadesse[1], Liyew Mekonen[2], Metsihet Tariku[3] and Semehal Haile[4]

Abstract

Background: Partograph is a simple, inexpensive & economical tool that provides a continuous graphical overview of labour and prevents prolonged and obstructed labor. The purpose of study is We aimed to assess partograph utilization and associated factors among obstetric care givers in governmental health institutions of Jigjiga and Degehabur Towns, Somali Region, Ethiopia.

Methods: An institution based cross-sectional quantitative study was carried out among obstetric care givers who were working in governmental health institutions. Systematic random sampling with proportional to size allocation was used to recruit a total of 235 study participants. Self-administered questionnaire was used to collect data in this study. Four health assistant lecturesThree data collectors and one supervisor were recruited and trained to facilitate the data collection activities. Data were entered into Epi data software and exported into SPSS (23.0) for analysis. Descriptive statistics, bivariate and multivariate logistic regression were computed to determine proportions and significant association with partograph utilization among obstetric care givers.

Result: Less than half of obstetric care givers, 41 %(95%CI: 34.5- 46.9) had good partograph utilization to monitor progress of labor. Being female[AOR =2.364, 95%CI:(1.027-5.441)], availability of partograph[AOR=4.633, 95%CI: (1.698-12.640)], having good knowledge [AOR=6.902, 95%CI:(2.620-18.183)], receiving on job training [AOR= 15.463, 95%CI:(6.948-34.416)] and positive attitude towards partograph [AOR=2.986, 95%CI:(1.250-7.136)] were significantly associated with partograph utilization.

Conclusion: Partograph utilization in this study was low. Especial emphasizes and interventions should be given to periodic on job training that improve knowledge and attitude of obstetric care givers to increase partograph utilization.

Key words: Partograph, Utilization, Obstetric care givers, Governmental health institutions, Somali Region, Ethiopia

[1]Department of Midwifery, College of Medicine and Health Sciences, Jigjiga University

[2]Department of Public Health, College of Medicine and Health Sciences, Jigjiga University

[3]Department of ~~Midwifery~~Nursing, College of Medicine and Health Sciences, Jigjiga University

[~~4~~3]Department of ~~Midwifery~~Neonatal Nursing, College of Medicine and Health Sciences, Jigjiga University

Background

Women are suffering many health problems during their pregnancy as well as at the time of child birth/delivery; globally, there were an estimated number of 15% expected births that developed life-threatening complications during pregnancy, delivery or the postpartum period (1-4). ~~In 2015 an estimated 303,000 women died because of pregnancy and childbirth-related complications. Of these, sub-Saharan Africa (66%) and Southern Asia (22%) accounted for 88% of the global burden (1, 2, 5-8).~~ In 2017, about 295 000 women died during and following pregnancy and childbirth. Sub-Saharan Africa alone accounted for roughly two-thirds (196 000) of maternal deaths, while Southern Asia accounted for nearly one-fifth (58 000); and both accounted for approximately 86% (254 000) of the estimated global maternal deaths(5).According to the 2015 Ethiopia Demographic and Health Survey, maternal mortality ratio was 353 per 100,000 live births in Ethiopia (8-11). Though it was slightly increased to 412 in 2016(12). Maternal mortality was still high in 2019 accounted to 412 per 100,000 live births(13).

The major causes of maternal and neonatal death in Ethiopia are obstructed labour and prolonged labour which was account for (13%) of maternal deaths (8, 14). Obstructed and prolonged labour contribute to series of complication in mothers and newborn infants if it is not managed early(15). These complications can be prevented by proper utilization of partograph during the progress of labour and providing skill delivery services ~~This complication can be prevented by accessing skilled delivery services such as proper utilization of partograph during the progress of labour~~(16).

The partograph is a graphical presentation of the progress of labour, and of fetal and maternal condition during labour ~~Partograph is an economical tool designed to provide a continuous pictorial overview of labour and prevent prolonged labour~~ (2, 6, 7, 14, 17). It consists of four sections: the maternal information, the fetal conditions record, the labor progress record, and the maternal conditions record (7, 18, 19). The fetal condition record may track fetal heart rate, amniotic liquor, and molding of the fetal skull. The labor progress record tracks cervical

dilatation and descent of the fetus' head over time, comparing it to a pre-printed "alert" and "action" lines (2, 8, 17, 18). The maternal conditions record often captures contractions, blood pressure, pulse, urine output, temperature, and drugs administered including drugs to help the uterus contract (7, 14, 18-20).

Universal utilization of the partograph during labour is recommended by world health organization since routine use of partograph is helpful to make better decisions for the diagnosis and management of prolonged and obstructed labour ~~The World Health Organization recommends the universal utilization of the partograph during labour and routine use of partograph is helpful to make better decisions for the diagnosis and management of prolonged and obstructed labour~~(7) . It is still not broadly used in the developing countries especially in Africa include Ethiopia. Studies done in Baghdad, a metropolitan area in Ghana and Lusaka: in Zambia, showed that58%, 54% and 87.5% of the participants used partograph to monitor progress of labour respectively (21-23). In Ethiopia, there is no consistent use of the partograph during labour; Studies done in Asella referral and teaching hospital, Sidama zone, Bale zone, East Gojjam zone, Addis Ababa city administration; showed that 26%, 50.7%, 70.2%, 53.85% and 69% of the participants used partograph to monitor progress of labour respectively(1, 6, 8, 16, 17). This inconsistent use of partograph due to different factors such as participant's sex, age, year of clinical service, health profession, knowledge about partograph, attitude towards partograph, absence of training on partograph, work place and work over load(1, 2, 7, 14, 16, 17, 21, 24).

Assessing obstetric care giver's practical utilization of partograph and its determinants has great value to design appropriate intervention strategies to provide quality maternity care. Therefore, the ~~aim~~ purpose of this study was to assess utilization of partograph and associated factors among obstetric care givers working in governmental health institutions of Jigjiga and Degehabur towns, Somali region, Ethiopia.

Methods

Study setting and period

The study was conducted in public health institutions Jigjiga and Degehabur towns from May18/2020 – July 8/2020. Jigjiga town, a capital city of Somali Region, is located 635kms east of Addis Ababa. It has 20 kebeles. The town administration has two hospitals, two health centres and fourteen health posts. In the town there are 1,208 clinical health professional workers (212 midwives, 11 HO, 137 medical doctors and 6 Gynecologist and obstetrician) that work in obstetric care at governmental health institutions. It has 366 total numbers of obstetric care givers.

Degehabur is a town in the Somali Region of Ethiopia, is located 800 &165 kms north of Addis Ababa and Jigjiga town respectively. It is the administrative centre of Degehabur woreda. The town administration has 1 hospital and 1 health centre of public health institutions. It has also 258 clinical health professional workers. Among those 51 midwives, 9 Health officer , 10 medical doctors, 1 Emergency Surgery and 1 obstetrician that work in obstetric care in the town of public health institutions. It has 72 total numbers of obstetric care givers. Total number of obstetric care givers in two towns is 438 that work public health institutions.

Study design ~~and~~

An institution based cross-sectional study design was conducted to assess utilization of partograph and associated factors.

Study population

All obstetric care providers (midwives, nurses, general practitioners, and health offers working in governmental health institutions) in Jigjiga and Degehabur Towns, Somali Region, Ethiopia included in the study. ~~Health professionals (Gynecologist and Obstetrician, students) and who did not attend labor cases were excluded from participating in the study.~~

Sample size determination

Formatted: Left

The sample size was estimated using single proportion formula by assuming 5% marginal error and 95% confidence interval (σ= 0.05) and study conducted in central zone, Tigray in which the proportion of utilization of partograph among obstetric care givers were73.3% (2). By adding 10% for non-respondents the final sample size was taken as 196. ~~It also estimated using double population proportion formula by assuming 95% CI, 80% power of the study, ratio of unexposed~~

to exposed 2.6 and AOR of 2.4 was used and the estimate proportion of obstetric care givers knowledge about partograph was highest sample size from others variables(7). By adding 10% of non-response rate, a total sample size was 235 (Table 1). This sample size was the final estimated sample size in this study which was higher than to the sample size calculated using single population proportion formula (Table 1). Moreover, the double population proportion formula was used to determine the sample size for factors associated with partograph utilization to get maximum possible sample size. It was calculated for some of the associated factors obtained from different literatures using Epi info statistical software version 7 with the following assumptions: confidence level = 95%, power = 80%, the ratio of unexposed to exposed almost equivalent to 2 and partograph utilization among those do not get additional training is 56.5% with AOR 2.4(14).Therefore, the final estimated sample size is 235 after adding 10% non response rate which was higher than the sample size calculated using single population proportion formula.

Sampling procedure

All health institutions found in Jigjiga and Degehabur towns were included and 6 health institutions were selected using simple random sampling technique. The total sample size of the study was distributed over each of the institute proportional to their size and the required number of study subjects was selected systematically from each selected governmental health institutions.From all health institutions found in Jigjiga and Degehabur towns, six health institutions (Jigjiga university Sheik Hassen Yabare Referal Hospital, Kharamara Hospital, Ablele health center, Ayerdega health center, Degehabour General Hospital, and Degehabour health center) were selected using a simple random sampling technique.

The total number of health professionals found in selected hospitals and health centers in Jigjiga town were 234 and 50 respectively. Additionally, 51 health professionals from the Degehabour General hospital and 21 from Degehabour health center were included. The required number of study subjects was calculated via proportional allocation, with 187 from Jigjiga and 48 from Degehabour. Study subjects were chosen using a simple random sampling procedure.

Data collection instrument and techniques

Semi - structured questionnaire which was developed from different literatures was used(2, 16, 17, 21, 22, 24). It was prepared in English version. Three data collectors and one supervisor were hired during the data collection period.

Data processing and analysis

Data was entered, checked, and analyzed using SPSS version 23.0. Descriptive statistics was employed to calculate frequencies, median and percentage. Attitude was measured as follows: there were six (6) attitude determining Likert scale type questions prepared to assess respondents' attitude towards partograph. The responses of "strongly agree was scored 5, agree was scored 4, uncertain was scored 3, disagree was scored 2 & strongly disagree was scored 1. The scoring was reversed for negative statements. The scores of the items were summed-up and the total was divided by the number of the items, giving a mean score. These score was converted into a percent score and mean were computed. The attitude was considered "positive" if the scored 60% or more and "negative" if less than 60%.

-Bivariable logistic regression analysis was made using OR and 95% CI to assess the association of independent variable with the outcome separately. Based on Bivariate analysis variables that showed significant association at ($p < 0.2$) were entered to multivariable analysis to select Predictor variables of factors affecting partograph utilization. Finally, variables that showed significant association at ($p < 0.05$) were identified as independent predictors of partograph utilization.

Ethical considerations

The Ethical approval was obtained from Institutional Review Board of College of Health Sciences, Jigjiga University on May 14/2020 with reference number RERC/016/2020. Communications with relevant bodies was made through a formal letter obtained from regional health. The objective and importance of the study was explained to the study participants. Data was collected after full informed written consent was obtained from participants. Confidentiality of the information and privacy is maintained.

Results

Socio-demographic characteristics of study participants

Out of 235 study participants, 228 Two hundred twenty eight respondents completed all the questionnaires correctly making a response rate of 97.02%. About 161 (70.6%) of obstetric care givers were females. The median age of the respondents were 26.00 (±3 IQR) years and more than half 128(56.1%) of them were within the age group 25-29 years. Regarding their profession, 152 (66.7%) were midwives followed by medical practitioner (MD) 31(13.6). Regarding to participant's working place, 162 (71.1%) of them were working at hospitals and the rest 57(25.0%) were working in health centers. More than two third of the respondents (68.4%) had ≤4 clinical service years and median year of clinical service of obstetric care givers were 3.00 (±3 IQR) years (Table 2).

Knowledge of obstetric care givers about partograph

From 228 participants, all of them (100%) had heard about partograph while 120(52.6%) of them know the exact definition of partograph. All participants had heard about partograph while 52.6% of them know the exact definition of partograph. Regarding to starting time of plot on partograph, 190(83.3%) of the participants correctly identified the exact time when to start plotting partograph. However, 148(61.4%) of the respondents did not know the component of partograph and also 71.5%, 68%, 76.8% of the respondents did not know the definition of alert line, action line and satisfactory of labour progress respectively. Pertaining to use of partograph, most of the participant 180 (78.9%) mentioned that partograph should be used for all women in active first stage of labor. From 228 participants, 145(63.6%) of the respondents did not take on job training related to partograph Majority (63.6%) of respondents (Table 23).

Regarding respondents' knowledge about partograph, only less than one third of them (28.5%) had good knowledge (Figure 3).

Attitude of obstetric care givers towards partograph utilization

From 228 participants, 105(46%) of them were Less than half of them (46%) strongly agreed that the partograph is beneficial. More than one third of participants, 103 (45.2%) of them

were agreed that partograph is favorable as it alerts obstetric care givers of any deviation from normal and 100(43.9%) of the respondents agreed that health care givers are able to identify problems and recognize complications early. From 228 respondents, 60(26.3%) Less than one third (26.3%) of them agreed that skilled birth attendant must use a partograph for every labouring mother. Out of 228 respondents, 70(30.7%) Near to thirty one percent (30.7%) of them were uncertain and 65(28.5%) were strongly disagreed that using partograph enables health care givers perform essential basic interventions. And also 80 (35.1%) of them were strongly disagreed that using partograph misleads management as the progress of labour and the partograph alert line are not aligned in most pregnant women(women (Table 34).

Table 1: Attitude of obstetric care givers towards partograph utilization governmental health institutions of Jigjiga and Degehabur Towns, Somali region, Ethiopia, 2020 (N=228)

Variables	Frequency	Percent
**Partograph is beneficial**		
Strongly agree	105	46
Agree	95	41.7
Uncertain	13	5.7
Strongly disagree	15	6.6
**Partograph is very favorable as it alerts health workers any deviation from normal**		
Strongly agree	50	22
Agree	113	49.6
Uncertain	45	19.7
Disagree	14	6.1
Strongly disagree	6	2.6
**By using partograph, health care providers are able to identify problems and recognize complications early**		
Strongly agree	31	13.6
Agree	100	43.9
Uncertain	70	30.7
Disagree	14	6.1
Strongly disagree	13	5.7
**Skilled birth attendant must use partograph on every laboring mother**		
Strongly agree	26	11.4
Agree	60	26.3
Uncertain	90	39.5
Disagree	39	17.1
Strongly disagree	13	5.7

**Using partograph enables health care givers perform essential basic interventions and make referrals to appropriate levels of care when necessary**		
Strongly agree	10	4.4
Agree	23	10.1
Uncertain	70	30.7
Disagree	60	26.3
Strongly disagree	65	28.5
**Using partograph misleads management as the progress of labour and the partograph alert line are not aligned in most pregnant woman**		
Strongly agree	16	7
Agree	22	9.6
Uncertain	50	21.9
Disagree	60	26.3
Strongly disagree	80	35.1

Regarding respondents' attitude towards partograph utilization, only less than one third of them (18%) had positive attitude (Figure 4).

Reasons for not using the partograph

From 228 respondents; 97(42.5%) of them were side that lack of orientation was the reason for not using the partograph when monitoring a women in labor (Figure 5)As indicated in figure 5, less than half (42.5%) of respondents said that lack of orientation was the reason for not using the partograph when monitoring women in labor.

.

Partograph utilization

In this study, majority (74.1%) of the respondents had reported the availability of partograph in the facility. More than half (64%) of the participants were partially recorded parameters of partograph on the tool. Similarly, (26.8%) of the participants were conducted deliveries from 7 to 9 in the institution per shift day. Most (92.1%) of the participants reported that they hadn't faced any problem to utilize partograph. Majority (68.4%) of participants were had work load in their facility. From 228 participants, (76.3%) More than three-fourths (76.3%

were) -gave advice for the obstetric care givers to encourage use partograph during labor (Table 45).

Overall from 228 participants, 93(41%) of the participants were had good partograph utilization (Figure 6).

Factors associated with partograph utilization

Bivariate analysis was performed to select candidate variables for multivariable analysis. Accordingly, sex of participant (being female)[COR=2.149, 95%CI: (1.162-3.975)], working in Hospital[COR=1.714, 95%CI: (0.937-3.135)], having good knowledge [COR=2.417, 95%CI: (1.244-4.696)], availability of partograph [COR= 6.451, 95%CI: (2.887-14.414)], taking on job training[COR=12.075, 95%CI: (6.343-22.988)] and positive attitude towards partograph [COR=2.734, 95%CI: (1.365-5.477)] were among factors showed that candidate variables for multivariate analysis at p value</= 0.2.

Multivariate analysis was performed for the purpose of controlling the multiple confounding effects of variables. Accordingly, sex of participant (being female) [AOR =2.364, 95%CI:(1.027-5.441)], availability of partograph[AOR=4.633, 95%CI: (1.698-12.640)], having good knowledge [AOR=6.902, 95%CI:(2.620-18.183)], receiving on job training [AOR=15.463, 95%CI:(6.948-34.416)] and positive attitude towards partograph [AOR=2.986, 95%CI:(1.250-7.136)] were factors showing significant association with partograph utilization at p-value</= 0.05 (Table 6).

DISCUSSION

The proportion of partograph utilization was 41% (95%CI: 34.6-46.9). This finding is inconsistent with study conducted in Baghdad (58%), Metropolitan area in Ghana (54%), Bale zone (70.2%), North Showa Zone (81.1%) , West Showa Zone (84.6%) and Central Tigray Zone (73.3%) (2, 6, 7, 14, 21, 22). The lack of well-designed and integrated programs, such as mentorship and supportive supervision, could be the cause of this difference. Other factors that may contribute to lower partograph utilization include a lack of expertise, a lack of understanding, insufficient partograph training, and a negative attitude among study participants.The possible reason for this variation could be due to poor availability of well-designed and coordinated programs like; the strength of mentorship, supportive supervision.

Other reasons might be competency level, poor knowledge, insufficient trainings provided on partograph and negative attitude of the study participants that decrease utilization of partograph. This finding is higher than the study conducted in Asella, Teaching and Referral Hospital, Ethiopia (1). These discrepancies may be due to the sample size (as sample size increase; it may increase rate of respondents, decrease effect estimate of a parameter and power of the tests), different study area and time of study (as time of study change (recent) there is change of strategies and plans to use partograph in the facility and stakeholders); that increase utilization of partograph. These discrepancies may be due to the sample size, different study area and time of study.

Findings of this study also indicated that positively significant association between sex and partograph utilization. Female obstetric care givers were more than two times more likely to good partograph utilization than males. This might be due to females are closer to obstetric information as they have a tendency to become midwives which makes more likely to have good knowledge of components of the partograph and favorable attitude towards partograph to utilize it than males. This finding may be entry point for health institutions and other stake holders to work on how to increase male involvement in partograph utilization. This finding is consistent with a previous study done in Bale zone, Ethiopia (6). It is understandable that the majority of birth attendants assigned to the labor ward on the study area are female midwives who have had the opportunity to participate in partograph usage trainings, which have increased their knowledge and abilities. But it is higher than the study conducted in Asella, Ethiopia and Central Tigray Zone (1, 2). These discrepancies may be due to study participants, since most of the participants are female in this study area as females are have more closer to obstetric care than males that influence partograph utilization.

Availability of the partograph in health facility is also associated with the toolits utilization. Those health care givers who had partograph tools in their institution were more than four times more likely to use partograph than had not partograph in their facility. This implies that the availability of the tools is necessary for the utilization of the tools to monitor the progress of the labour. This finding is consistent with a previous study done in Asella, Ethiopia and West Showa Oromia, Ethiopia (1, 14). This implies that the availability of partograph by

itself is essential to motivate obstetrics giver for use of this tools starting from active first stage of labour.

Findings of this study also indicated that significant association between knowledge and partograph utilization. Those obstetric care givers who had good knowledge about partograph were more than six times more likely to good partograph utilization than their counterparts. This finding is consistent with a previous study conducted in Enugu Metropolis, North Showa Zone, Ethiopia, West Showa Zone, Oromia, Ethiopia and Bale zone(6, 7, 14, 24). , but this finding is higher than others study conducted in Addis Ababa city administration and East Gojjam zone (8, 16). This discrepancy might be due to different in place and time of the study that may be explained with different strategies in partograph utilization, different level of attitude of obstetric care givers towards partograph and insufficient training about partograph; This enables them to understand components of partograph, use of partograph, increase attitude towards partograph, what critical progress of labour will occur and decide on alternatives such as referral and caesarian section. The possible reason might be Obstetrics care givers who have knowledge on purpose of partograph may use it more frequently to prevent prolonged labour and related

Incomplications.

this study; findings This research finding showed that on-job training on partograph had a significant association with partograph utilization. Those obstetric care givers who received on job training on partograph were more than fifteen times more likely to utilize partograph than who had not received on- job training. This observation could be explained by the fact that, obstetric care givers who received training had better exposure to practice about partograph utilization, better be able to explain components of the partograph, better to have good knowledge about partograph and better to have positive attitude towards partograph that in turn improves their partograph utilization. This finding is supported by the study conducted in West Showa zone Oromia Ethiopia, Addis Ababa city administration, Bale zone, East Gojjam zone, central Tigray zone, Easter Ethiopia, North Showa zone and Sidama zone (2, 6-8, 14, 16, 17, 20).; but this finding is inconsistent with studies conducted in Baghdad ,Wolaita zone and Asella referral hospital (1, 5, 21). The reason may due to different study area and time; since as study area different and time goes on, there is a change in policy, strategy and

implementation of training on partograph that increase or decrease its utilization. On job trainings enhance knowledge ; and improve attitude and skills which in turn increase partograph utilization

Findings of this study also indicated that significant association between attitude of participants towards partograph and partograph utilization. Those obstetric care givers who had positive attitude towards partograph were three times more likely to utilize partograph than to their counterparts. This could be due to the fact that, having a positive attitude towards partograph utilization might come after having knowledge about partograph that may influence the utilization of partograph. This finding is consistent with a previous study done in North Showa Zone, Ethiopia (7).; but this finding is inconsistent with studies done in Baghdad, Enugu metropolis, Central Tigray zone, Asella referral hospital, West Showa zone, East Gojjam zone and Bale zone (1, 2, 6, 8, 14, 21, 24). The reason may due to different study area and time, having different knowledge and receive on job training; since having good knowledge and receive on job training that may change the attitude of participants towards partograph that influence utilization of partograph. The possible reason might be those who had good attitude toward the use of partograph might be committed to improve their skill in use.

The strength of this study was that it gave equal chance to all participants since it used systematic random sampling method. Limitations of this study; cross sectional study design by its very nature cannot establish cause and effect relationship. Since information about partograph utilization was obtained from respondents through self administered questionnaires, rather than observation; recall bias, response bias and social desirability bias are the potential limitations of this study. However, numerous scientific procedures were employed to minimize the possible effects. To reduce the response bias, for instance, the aim of the study was discussed with respondents in order to obtain genuine response. In addition, procedures such as supervision, pretest of data collection tool and adequate training of data collectors and supervisor were utilized. Small sample size hampers the precion of some associations and may affect estimate of a parameter and power of the test. Inclusion of private health care providers would have given comprehensive picture and make generalization possible.

CONCLUSION AND RECOMMENDATIONS

This study revealed that the proportion of partograph utilization by the study participants was low according to Modified WHO partograph and previous studies. Being female obstetrics care giverSex (being female), availability of partograph, having good knowledge about partograph, having on job training and having positive attitude of participants towards partograph were positively significant associated with partograph utilization.

To Jigjiga and Degehabur towns health offices

Sustainable and accessible supply of partograph sheets should be available to all institutions to maintain consistency of its utilization.

To stake holders

Provision of periodic on-job training on partograph is mandatory to improve knowledge and attitude of obstetric care givers towards partograph utilization. To increase male involvement in partograph utilization; health institutions and other stake holders to work on how should be give special training for male that create awareness, to change their attitude and employee male professionals.

To obstetric care givers

Partograph should be used for all labouringlaboring women and taken seriously by the care givers and it should be considered as a tool for diagnosing problems, perform essential basic interventions and make referral to appropriate levels of care when necessary during the progress of labour. Update their knowledge and attitude towards partograph utilization. A great commitment on utilization of partograph is very necessary.

To researchers

Further researchers have to be done using qualitative research methodology and on private health institutions to get a comprehensive picture.

~~Further researchers will include qualitative research methodology and private health institutions to given comprehensive picture and make generalization possible.~~

Abbreviations

ANC: Ante Natal Care; AOR: Adjusted Odds Ratio; APGAR: Activity, Pulse, Grimace, Appearance &Respiration; CI: Confidence Interval; COR: Crude Odds Ratio; CSA: Central Statistical Agency; EDHS: Ethiopian Demographic and Health Survey; FHR: Fetal Heart Rate; ICM: International Council of Midwives; IMB: Integrated Model of Behavior; MCH: Maternal and Child Health; MM: Maternal Mortality; MMR Maternal Mortality Rate; SD: Standard Deviation; SMI: Safe Motherhood Initiatives; SPSS: Statistical Package for Social Sciences; UN: United Nation; UNFPA: United Nations Population Fund Affairs; UNICEF: United Nations Children Fund; USA: United State of America; WHO: World Health Organization

Acknowledgment

We appreciate Jigjiga University, College of medicine and Health Sciences, School of Graduate Studies, department of Maternity Nursing for giving us this opportunity. We would like to thank Karamara hospital administration office, Jigjiga University Shuik Hassan Yabare Referral Hospital, Jigjiga City health office and Degehabur town health office staffs; for giving base line information for this study. Finally, we would like to thank data collectors for the contribution of data collection and we would like to thank participates that voluntary involve in this study. We would also like to extend our appreciation to those who has helped me a lot in giving additional advice.

Authors' contributions

AT designed the research, analyzed the data, interpreted the findings and drafted the manuscript. LM and MT involved in all process of this research.SH reviewed a manuscript. All authors read and approved the final manuscript for publication.

Funding

Not applicable.

Availability of data and materials

The datasets generated and/or analyzed during the current study are available from the corresponding author on reasonable request. The datasets generated and/or analyzed during the current study are fully available.

Consent for publication

Not applicable.

Competing interests

There is no competing interest among the authors.

Author details

[1]College of Medicine and Health sciences, department of Midwifery, Jigjiga University, Jigjiga, Ethiopia. [2] College of Medicine and Health sciences, department of Midwifery, Jigjiga University, Jigjiga, Ethiopia. [3] College of Medicine and Health sciences, department of Midwifery, Jigjiga University, Jigjiga, Ethiopia

References

1. Bekele D, Beyene K, Hinkosa L, Shemsu MN. Partograph Utilization and Associated Factors among Graduating Health Professional Students in Asella Referal and Teaching Hospital, Ethiopia, 2016. STM Journal. 2016;6(2).
2. Hailu T, Nigus K, Gidey G, Hailu B, Moges Y. Assessment of partograph utilization and associated factors among obstetric care givers at public health institutions in central zone, Tigray, Ethiopia. BMC Res Notes. 2018;11(1):710.
3. Melese KG, Bekiru ET. Knowledge of partograph utilization and its associated factors: A cross-sectional survey in Wolaita Zone Southern Ethiopia. International Journal of Nursing and Midwifery. 2019;11(1):1-6.
4. Muthusi UM, Nyamoita MG, Stephen M. Contribution of Completed Modified World Health Organization Partograph on Maternal and Foetal Mortality Reduction in Health Facilities in Makueni County, Kenya. Health. 2019;5(6):227-36.

5. Bekiru KGMaET. Knowledge of partograph utilization and its associated factors: A cross-sectional survey in Wolaita Zone Southern Ethiopia. International Journal of Nursing and Midwifery. January 2019;Vol. 11(1):pp. 1-6.
6. Markos D, Bogale D. Knowledge and utilization of partograph among health care professionals in public health institutions of Bale zone, Southeast Ethiopia. Public health. 2016;137:162-8.
7. Wakgari N, Amano A, Berta M, Tessema GA. Partograph utilization and associated factors among obstetric care providers in North Shoa Zone, Central Ethiopia: a cross sectional study. Afr Health Sci. 2015;15(2):552-9.
8. Zelellw DA, Tegegne TK. Level of partograph utilization and its associated factors among obstetric caregivers at public health facilities in East Gojam Zone, Northwest Ethiopia. PloS one. 2018;13(7):e0200479.
9. Alkema L, Chou D, Hogan D, Zhang S, Moller A-B, Gemmill A, et al. Global, regional, and national levels and trends in maternal mortality between 1990 and 2015, with scenario-based projections to 2030: a systematic analysis by the UN Maternal Mortality Estimation Inter-Agency Group. The Lancet. 2016;387(10017):462-74.
10. Federal Democratic Republic of Ethiopia Ministry of Health (FMOH) EPHI. National maternal death surveillance and response annual report 2008 EFY. FMOH, EPHI. 2017.
11. WHO U, UNFPA, and The World Bank and the United Nations Population Division. Trends in Maternal Mortality. World Health Organization. 2015.
12. Agency CS. Census, in 2015. Central Statistical Agency and ICF International. 2015.
13. Indicators K. Mini Demographic and Health Survey. EPHI and ICF. 2019.
14. Willi W. Magnitude of Partograph Use and Factors that Affecting Its Utilization Among Obstetric Caregivers in Public Health Institutions of West Showa Zone, Oromia Regional State, Ethiopia 2015: Addis Ababa University; 2015.
15. Bosomprah S, Tatem AJ, Dotse- Gborgbortsi W, Aboagye P, Matthews Z. Spatial distribution of emergency obstetric and newborn care services in Ghana: Using the evidence to plan interventions. International Journal of Gynecology & Obstetrics. 2016;132(1):130-4.
16. Hagos AA, Teka EC, Degu G. Utilization of Partograph and its associated factors among midwives working in public health institutions, Addis Ababa City Administration, Ethiopia, 2017. BMC Pregnancy and Childbirth. 2020;20(1):49.

17. Kidist Eshetu EH, Dubale Dulla. Magnitude of Partograph Use and Associated Factors among Obstetric Care Givers in Public Health Institution in Sidama Zone, Southern Ethiopia. Diversity and Equality in Health and Care (2017) 14(6): 316-3232017;14(6):316-23.

18. Angasu K. Assessment of partograph utilization and associated factors among obstetric care providers in public health facilities in Jimma town, Jimma zone, Oromia region , South West Ethiopia, 2016. BMC Pregnancy Childbirth 2016;60(2).

19. Chemeda G, Teklewold B, Daka K. Partograph Utilization and Factors Associated with Poor Perinatal Outcomes in Wolaita Sodo University Referral Hospital, Southern Ethiopia. Journal of Health, Medicine and Nursing. 2019;63.

20. Mezmur H, Semahegn A, Tegegne BS. Health professional's knowledge and use of the partograph in public health institutions in eastern Ethiopia: a cross-sectional study. BMC Pregnancy Childbirth. 2017;17(1):291.

21. Abdul-Saheb TM, Al-Deen LD. Knowledge and Use of Partograph among Obstetric Care Givers in a Sample of Hospitals in Baghdad-2017. Iraqi Journal Of Community Medicine 2017;30(3):115-22.

22. Opoku BK, Nguah SB. Utilization of the modified WHO partograph in assessing the progress of labour in a metropolitan area in Ghana. Research Journal of Women's Health. 2015;2(2):1-7.

23. Victoria Mwiinga Kalusopa SM, Mutinta Muleya, Kutemba Lyangenda, Maureen Sianga, Francesca Msiska, Carol Porteous, Concepta Kwaleyela, Alice Ngoma Hazemba and Margaret Maimbolwa. Use of the partograph among midwives at University Teaching Hospital, Lusaka: a LAMRN project in Zambia. African journal of midwifery and women's health. 2017;11(2).

24. Nwaneri A, Ndie E, Ehiemere I, Okafor E, Ezenduka P. Evaluation of Factors Affecting the Utilization of Partograph by Nurses/Midwives in Primary/Secondary Health Facilities in Enugu Metropolis. Gynecol Obstet (Sunnyvale). 2017;7(446):2161-0932.1000446.

Response to editor:

1. I prepare manuscript based on PLOS ONE's style requirements
2. Please include additional information regarding the survey or questionnaire
    - “Semi - structured questionnaire was developed reviewing different literatures (2, 16, 17, 21, 22, 24).
    - References are found in the manuscript.
3. In your Data Availability statement, you have not specified where the minimal data set underlying the results described in your manuscript can be found.
    - ✓ The datasets generated and/or analyzed during the current study are fully available.
    - ✓ We will attach via SPSS file.
4. Please include your tables as part of your main manuscript and remove the individual files. Please note that supplementary tables (should remain/ be uploaded) as separate "supporting information" files.
    - ✓ Comment accepted and modified accordingly.
5. Please revise the manuscript to rephrase the duplicated text, cite your sources, and provide details as to how the current manuscript advances on previous work.
    - ✓ Comment accepted and modified accordingly

Response to Reviewer 1 comments:

1. What do you mean by four assistant lectures?
    - ✓ Those are midwives who are working in teaching institution
2. Observe spacing in the whole document
    - ✓ I Accept your comment and correct the document accordingly
3. Spacing
    - ✓ I Accept your comment and correct the document accordingly
4. Show the formula you used to find the proportion, what was the total number of HCPs in all health facilities?
    - ✓ From all health institutions found in Jigjiga and Degehabur towns, six health institutions (Jigjiga university Sheik Hassen Yabare Referal Hospital, Kharamara Hospital, Ablele health center, Ayerdega health center, Degehabour General Hospital, and Degehabour health center) were selected using a simple random sampling technique.The total number of health professionals found in selected hospitals and health centers in Jigjiga town were 234 and 50 respectively. Additionally, 51 health professionals from the Degehabour General hospital and 21 from Degehabour health center were included. The required number of study subjects was calculated via proportional allocation, with 187 from Jigjiga and 48

from Degehabour. Study subjects were chosen using a simple random sampling procedure.

5. Did they have a training concerning data collection? if yes for how long?
    - ✓ Three data collectors and one supervisor were trained for two days and hired during the data collection period.
6. How was attitude analyzed?
    - ✓ **Attitude towards partograph:** Attitude is the obstetric care givers opinion, outlook, position and ideas they have toward partograph and its' utilization.
    - ✓ **Attitude scale***:* There were six (6) attitude determining Likert scale type questions prepared to assess respondents' attitude towards partograph. The responses of "strongly agree was scored 5, agree was scored 4, uncertain was scored 3, disagree was scored 2 & strongly disagree was scored 1. The scoring was reversed for negative statements. The scores of the items were summed-up and the total was divided by the number of the items, giving a mean score. These score was converted into a percent score and mean were computed. The attitude was considered "positive" if the scored 60% or more and "negative" if less than 60%.
    - ✓ **Positive Attitude**: Obstetric care givers who scored mean value and above to attitude related questions. It was measured by using a 5-point Likert scale as individuals responded strongly agree and agree for favorable attitude was given score of four (4) and five(5).
    - ✓ **Negative attitude**: Score of one (1), two (2) and three (3) for those who was responded as strongly disagree, disagree and uncertain while the above scores were reversed for unfavorable attitude questions.
7. Add ethical clearance reference number and the date it was obtained
    - ✓ The Ethical approval was obtained from Institutional Review Board of College of Health Sciences, Jigjiga University on May 14/2020 with reference number RERC/016/2020.
8. This is not a scientific reason!!!
    - ✓ It is understandable that the majority of birth attendants assigned to the labor ward on the study area are female midwives who have had the opportunity to participate in partograph usage trainings, which have increased their knowledge and abilities.

Response to Reviewer 2 comments:

1. Instead of "We aimed" in the abstract, "the purpose of study is…" should be written in a more academic language.

- ✓ Corrected based on your comment

2. "In 2015 an estimated 303,000 women died because of pregnancy and childbirth-related complications." If there is a more up-to-date statistic, it would be more appropriate to use it.

- ✓ I incorporate updated data based on your comment:” In 2017, about 295 000 women died during and following pregnancy and childbirth. Sub-Saharan Africa alone accounted for roughly two-thirds (196 000) of maternal deaths, while Southern Asia accounted for nearly one-fifth (58 000); and both accounted for approximately 86% (254 000) of the estimated global maternal deaths”

3. The problem description is well written.
4. “Study design and” and should be removed

- ✓ Corrected

5. study population
… Health professionals (Gynecologist and Obstetrician, students) and who did not attend labor cases were excluded from … this is not an exclusion criterion, it can be removed.

- ✓ Corrected based on your comment

6. It is necessary to explain the sampling method with a more understandable and clear expression. In addition, it should be clearly stated how many people were included in the study in the Study Population.

We elaborate a sampling procedure in the document as follows:

- ✓ From all health institutions found in Jigjiga and Degehabur towns, six health institutions (Jigjiga university Sheik Hassen Yabare Referal Hospital, Kharamara Hospital, Ablele health center, Ayerdega health center, Degehabour General Hospital, and Degehabour health center) were selected using a simple random sampling technique.The total number of health professionals found in selected hospitals and health centers in Jigjiga town were 234 and 50 respectively. Additionally, 51 health professionals from the Degehabour General hospital and 21 from Degehabour health center were included. The required number of study subjects was calculated via proportional allocation, with 187 from Jigjiga and 48 from Degehabour. Study subjects were chosen using a simple random sampling procedure.

7. “Semi - structured questionnaire which was developed from different literatures was used.” The sources used should be stated at the end of the sentence.

- ✓ Corrected based on your comment accordingly

“Semi - structured questionnaire which was developed from different literatures was used (2, 16, 17, 21, 22, 24).”

8. Ethics committee decision number should be attached.

- ✓ I attached a letter as supplementary file.
  - ✓ The Ethical approval was obtained from Institutional Review Board of College of Health Sciences, Jigjiga University on May 14/2020 with reference number RERC/016/2020.

9. Table 1 is in the wrong place in the text.

- ✓ Modified based on your comment accordingly

10. Sociodemographic findings should be Table 1 and the findings should be cited in parentheses accordingly. Table 2 should not be started.

- ✓ Corrected accordingly

11. Results- Socio-demographic characteristics of study participants –
Out of 235 study participants, 228… This section should be specified in the number of samples in the method.

- ✓ Corrected based on your comment accordingly

12. In the findings, the sentence should not be started as “From 228 participants…”, a more academic language should be used.

- ✓ Corrected based on your comment accordingly

13. In the findings, it is sufficient to write only the percentages instead of the frequencies. There should be no need to use parentheses.

- ✓ Corrected based on your comment accordingly

14. In table 1 “Using partograph misleads management as the progress of labor and the partograph alert line are not aligned in most pregnant woman” percent sum is not 100. It should be corrected.

- ✓ Corrected based on your comment accordingly
- ✓ 7+9.7+21.9+26.3+35.1=100

15. In the discussion section, first the findings of the study and then the findings in the literature should be included. Commentary sentences should be written after the studies are compared. The spelling of some sections is appropriate, but some require editing.

- ✓ Corrected based on your comment accordingly

16. Suggestions should also be included in line with the results of the study. Missing suggestions should be added.

- ✓ Corrected based on your comment accordingly

17. In Table 2: “List one complication” percent sum is not 100.

- ✓ Corrected based on your comment accordingly
    - ❖ 96.8+8.2=100

18. In the table titles, “N” should be written as “n” in lower case.

    - ❖ Corrected based on your comment accordingly
