# Supplementary material for: Partograph utilization and associated factors among obstetric care givers in governmental health institutions of Jigjiga and Degehabur towns, Somali region, Ethiopia: A cross-sectional study
Source: PLoS One. 2022 Mar 9;17(3):e0264373. doi: 10.1371/journal.pone.0264373 (PMC8906604; doi:10.1371/journal.pone.0264373)
Supplement: Attachment — Submitted filename: Response to Editor & Reviewers.docx [file pone.0264373.s003.docx]

**Response to editor:**

1. I prepare manuscript based on PLOS ONE's style requirements
2. Please include additional information regarding the survey or questionnaire

- “Semi - structured questionnaire was developed reviewing different literatures (2, 16, 17, 21, 22, 24).
- References are found in the manuscript.

3. In your Data Availability statement, you have not specified where the minimal data set underlying the results described in your manuscript can be found.

- The datasets generated and/or analyzed during the current study are fully available.
- We will attach via SPSS file.

4. Please include your tables as part of your main manuscript and remove the individual files. Please note that supplementary tables (should remain/ be uploaded) as separate "supporting information" files.

- Comment accepted and modified accordingly.

5. Please revise the manuscript to rephrase the duplicated text, cite your sources, and provide details as to how the current manuscript advances on previous work.

- Comment accepted and modified accordingly

**Response to Reviewer 1 comments:**

1. What do you mean by four assistant lectures?

- Those are midwives who are working in teaching institution

2. Observe spacing in the whole document

- I Accept your comment and correct the document accordingly

3. Spacing

- I Accept your comment and correct the document accordingly

4. Show the formula you used to find the proportion, what was the total number of HCPs in all health facilities?

- From all health institutions found in Jigjiga and Degehabur towns, six health institutions (Jigjiga university Sheik Hassen Yabare Referal Hospital, Kharamara Hospital, Ablele health center, Ayerdega health center, Degehabour General Hospital, and Degehabour health center) were selected using a simple random sampling technique.The total number of health professionals found in selected hospitals and health centers in Jigjiga town were 234 and 50 respectively. Additionally, 51 health professionals from the Degehabour General hospital and 21 from Degehabour health center were included. The required number of study subjects was calculated via proportional allocation, with 187 from Jigjiga and 48 from Degehabour. Study subjects were chosen using a simple random sampling procedure.

5. Did they have a training concerning data collection? if yes for how long?

- Three data collectors and one supervisor were trained for two days and hired during the data collection period.

6. How was attitude analyzed?

- **Attitude towards partograph:** Attitude is the obstetric care givers opinion, outlook, position and ideas they have toward partograph and its’ utilization.
- **Attitude scale***:* There were six (6) attitude determining Likert scale type questions prepared to assess respondents’ attitude towards partograph. The responses of “strongly agree was scored 5, agree was scored 4, uncertain was scored 3, disagree was scored 2 & strongly disagree was scored 1. The scoring was reversed for negative statements. The scores of the items were summed-up and the total was divided by the number of the items, giving a mean score. These score was converted into a percent score and mean were computed. The attitude was considered “positive” if the scored 60% or more and “negative” if less than 60%.
- **Positive Attitude**: Obstetric care givers who scored mean value and above to attitude related questions. It was measured by using a 5-point Likert scale as individuals responded strongly agree and agree for favorable attitude was given score of four (4) and five(5).
- **Negative attitude**: Score of one (1), two (2) and three (3) for those who was responded as strongly disagree, disagree and uncertain while the above scores were reversed for unfavorable attitude questions.

7. Add ethical clearance reference number and the date it was obtained

- The Ethical approval was obtained from Institutional Review Board of College of Health Sciences, Jigjiga University on May 14/2020 with reference number RERC/016/2020.

8. This is not a scientific reason!!!

- It is understandable that the majority of birth attendants assigned to the labor ward on the study area are female midwives who have had the opportunity to participate in partograph usage trainings, which have increased their knowledge and abilities.

**Response to Reviewer 2 comments:**

1. Instead of “We aimed” in the abstract, “the purpose of study is…” should be written in a more academic language.

- Corrected based on your comment

2. “In 2015 an estimated 303,000 women died because of pregnancy and childbirth-related complications.” If there is a more up-to-date statistic, it would be more appropriate to use it.

- I incorporate updated data based on your comment:” In 2017, about 295 000 women died during and following pregnancy and childbirth. Sub-Saharan Africa alone accounted for roughly two-thirds (196 000) of maternal deaths, while Southern Asia accounted for nearly one-fifth (58 000); and both accounted for approximately 86% (254 000) of the estimated global maternal deaths”

3. The problem description is well written.
4. “Study design and” and should be removed

- Corrected

5. study population
… Health professionals (Gynecologist and Obstetrician, students) and who did not attend labor cases were excluded from … this is not an exclusion criterion, it can be removed.

- Corrected based on your comment

6. It is necessary to explain the sampling method with a more understandable and clear expression. In addition, it should be clearly stated how many people were included in the study in the Study Population.

We elaborate a sampling procedure in the document as follows:

- From all health institutions found in Jigjiga and Degehabur towns, six health institutions (Jigjiga university Sheik Hassen Yabare Referal Hospital, Kharamara Hospital, Ablele health center, Ayerdega health center, Degehabour General Hospital, and Degehabour health center) were selected using a simple random sampling technique.The total number of health professionals found in selected hospitals and health centers in Jigjiga town were 234 and 50 respectively. Additionally, 51 health professionals from the Degehabour General hospital and 21 from Degehabour health center were included. The required number of study subjects was calculated via proportional allocation, with 187 from Jigjiga and 48 from Degehabour. Study subjects were chosen using a simple random sampling procedure.

7. “Semi - structured questionnaire which was developed from different literatures was used.” The sources used should be stated at the end of the sentence.

- Corrected based on your comment accordingly

“Semi - structured questionnaire which was developed from different literatures was used (2, 16, 17, 21, 22, 24).”

8. Ethics committee decision number should be attached.

- I attached a letter as supplementary file.
- The Ethical approval was obtained from Institutional Review Board of College of Health Sciences, Jigjiga University on May 14/2020 with reference number RERC/016/2020.

9. Table 1 is in the wrong place in the text.

- Modified based on your comment accordingly

10. Sociodemographic findings should be Table 1 and the findings should be cited in parentheses accordingly. Table 2 should not be started.

- Corrected accordingly

11. Results- Socio-demographic characteristics of study participants –
Out of 235 study participants, 228… This section should be specified in the number of samples in the method.

- Corrected based on your comment accordingly

12. In the findings, the sentence should not be started as “From 228 participants…”, a more academic language should be used.

- Corrected based on your comment accordingly

13. In the findings, it is sufficient to write only the percentages instead of the frequencies. There should be no need to use parentheses.

- Corrected based on your comment accordingly

14. In table 1 “Using partograph misleads management as the progress of labor and the partograph alert line are not aligned in most pregnant woman” percent sum is not 100. It should be corrected.

- Corrected based on your comment accordingly
- 7+9.7+21.9+26.3+35.1=100

15. In the discussion section, first the findings of the study and then the findings in the literature should be included. Commentary sentences should be written after the studies are compared. The spelling of some sections is appropriate, but some require editing.

- Corrected based on your comment accordingly

16. Suggestions should also be included in line with the results of the study. Missing suggestions should be added.

- Corrected based on your comment accordingly

17. In Table 2: “List one complication” percent sum is not 100.

- Corrected based on your comment accordingly
- 96.8+8.2=100

18. In the table titles, “N” should be written as “n” in lower case.

- Corrected based on your comment accordingly
