# Supplementary material for: Partograph utilization and associated factors among obstetric care givers in governmental health institutions of Jigjiga and Degehabur towns, Somali region, Ethiopia: A cross-sectional study
Source: PLoS One. 2022 Mar 9;17(3):e0264373. doi: 10.1371/journal.pone.0264373 (PMC8906604; doi:10.1371/journal.pone.0264373)
Supplement: Attachment — Submitted filename: Response to Editor & Reviewers.docx [file pone.0264373.s005.docx]

**Response to Reviewer 1 comments:**

1. Author should put space between words and also she/he should put all decimal points into two, this should be done in the whole document.

**Response**: I accepted and modified the document according to your comments.

**Response to Reviewer 2 comments:**

1. In the result section: In table 1 “Using partograph misleads management as the progress of labor and the partograph alert line are not aligned in most pregnant woman” percent sum is not 100. It should be corrected.

**Response:** It is found in table 3 of the manuscript and the percent sum is 100 %( 7+9.7+21.9+26.3+35.1=100)

2. There are two tables called Table 1.

**Response**: It was corrected.

3. Suggestion statements should be added in the recommendation section.

**Response:** We took some modification

4. In Table 2: “List one complication” percent sum is not 100.

**Response:** Corrected based on your comment accordingly

- 91.8+8.2=100

5. Table 3 should be written, instead of table 4.

**Response:** We put table 3 and table 4 based on their logical order.

6. In the table titles, “N” should be written as “n” in lower case

**Response:** It was corrected based on previous comment
